# Relationship of polymorphisms in the tissue inhibitor of metalloproteinase (*TIMP*)-1 and -2 genes with chronic heart failure

**DOI:** 10.1038/s41598-018-27857-5

**Published:** 2018-06-21

**Authors:** Evelise Regina Polina, Raquel Rosa Candebat Vallejo Araújo, Renan Cesar Sbruzzi, Andréia Biolo, Luís Eduardo Rohde, Nadine Clausell, Kátia Gonçalves dos Santos

**Affiliations:** 1 Laboratory of Human Molecular Genetics, Universidade Luterana do Brasil (ULBRA), Canoas, 92425-900 Brazil; 2 Heart Failure and Cardiac Transplant Unit, Cardiology Division, Hospital de Clínicas de Porto Alegre (HCPA), Porto Alegre, 90035-903 Brazil; 3 Department of Internal Medicine, Medical School, Universidade Federal do Rio Grande do Sul (UFRGS), Porto Alegre, 90040-341 Brazil

## Abstract

Dysregulated expression of tissue inhibitors of matrix metalloproteinases (TIMPs) is associated with systolic dysfunction and worsening heart failure (HF). However, no study has assessed the relationship between *TIMP* polymorphisms and chronic HF. In this study, 300 HF outpatients with reduced left ventricular ejection fraction and 304 healthy blood donors were genotyped for the 372 T > C polymorphism (Phe124Phe; rs4898) in the *TIMP-1* gene and the −418 G > C polymorphism (rs8179090) in the *TIMP-2* gene to investigate whether these polymorphisms are associated with HF susceptibility and prognosis. The genotype and allele frequencies of the 372 T > C polymorphism in HF patients were not significantly different from those observed among healthy subjects, and the C allele of the −418 G > C polymorphism was very rare in our population (frequency < 1%). After a median follow-up duration of 5.5 years, 121 patients (40.3%) died (67 of them from HF). Survival analysis did not show statistically significant differences in all-cause death and HF-related death between patients with and without the T allele (*P* > 0.05 for all comparisons). Thus, our findings do not support the hypothesis that the 372 T > C (Phe124Phe) polymorphism in the *TIMP-1* gene and the −418 G > C polymorphism in the *TIMP-2* gene are associated with HF susceptibility and prognosis in Southern Brazilians.

## Introduction

Heart failure (HF) is a chronic and progressive syndrome of insufficient cardiac output resulting from myocardial injury; it remains a leading cause of morbidity and mortality worldwide^1^. HF is a multifactorial disease, and the interaction of several genetic variants results in differential HF susceptibility, therapeutic response, and prognosis^2^. During HF development, the left ventricle (LV) undergoes structural and functional changes involving cardiomyocyte death, fibrosis, inflammation, and electrophysiological remodelling^3,4^. This process of cardiac remodelling is mediated by the action of matrix metalloproteinases and their inhibitors^5–7^, resulting in the deterioration of cardiac function and progressive HF^8^.

Matrix metalloproteinases (MMPs) are a family of proteolytic enzymes that regulate extracellular matrix (ECM) turnover and inflammatory signalling^5^. Activated MMPs are tightly regulated by endogenous tissue inhibitor of metalloproteinases (TIMPs), which also exert their effects on cell proliferation, differentiation, apoptosis, and angiogenesis by MMP-independent mechanisms^9,10^. TIMP-1 inhibits proMMP-9, while TIMP-2 preferentially binds to proMMP-2^10^. In heart disease, the expression levels of MMPs and TIMPs are unbalanced, which may contribute to collagen disintegration in myocardial tissue and to alterations in cardiomyocyte intracellular signalling^9^. High levels of TIMP-1 in either the plasma or myocardium have been found in patients with hypertension^11^, myocardial fibrosis, LV hypertrophy, systolic and diastolic dysfunction, atrial fibrillation, acute myocardial infarction (AMI), end-stage idiopathic dilated cardiomyopathy, and progressive HF. Increased levels of TIMP-2 are also associated with systolic dysfunction, AMI, end-stage idiopathic dilated cardiomyopathy^9^, and acute kidney injury stage 2–3 in decompensated HF^12^. However, other studies have reported decreased levels of TIMP-2 in patients with coronary artery disease (CAD)^13^ and systolic HF^14,15^ and in those who died from or were admitted for HF following mitral valve surgery^16^.

Several functional polymorphisms identified in the *MMP* gene promoters have been found to be associated with clinical outcomes in patients with LV remodelling and failing hearts^6^. In a previous study, we showed that high serum levels of MMP-9 are associated with carotid plaque vulnerability and stroke in patients who underwent endarterectomy^17^. In other studies, we also observed a lower rate of HF-related death in HF patients who carried the 2 G allele of the −1607 1 G/2 G polymorphism in the *MMP-1* gene^18^, the TT genotype of the −790 G > T polymorphism in the *MMP-2* gene^19^, or the 6 A allele of the −1171 5 A/6 A polymorphism in the *MMP-3* gene^18^ than patients with other genotypes. Considering our previous findings and the role of MMPs and TIMPs in HF pathogenesis, we decided to expand our study by investigating the association of *TIMP* gene variants with HF.

TIMP-1 is located on the Xp11.23–11.4 chromosome, while TIMP-2 is located on the 17q23-25 chromosome^9,10^. The T allele of the 372 T > C silent mutation at exon 5 of the *TIMP-1* gene (Phe124Phe) is associated with spontaneous deep intracerebral haemorrhage in elderly Taiwanese males^20^, increased serum levels of TIMP-1, and a higher mortality rate at 30 days from intensive care unit admission in Caucasians with severe sepsis^21^. In the context of LV dysfunction, however, the 372 T > C polymorphism was not associated with AMI, CAD^22^, acute HF^23^, or adverse prognosis in patients with ST elevation myocardial infarction^24^. The C allele of the G > C transversion at nucleotide position −418 of the *TIMP-2* gene promoter is thought to down-regulate gene expression by abolishing the Sp1 binding site^25^. The C allele was associated with increased magnitude of QT and QTc dispersion prolongation in a healthy elderly Chinese cohort^26^, increased susceptibility to atrial fibrillation in Chinese Han patients with hypertensive heart disease, and reduced plasma levels of TIMP-2^27^. To the best of our knowledge, no study has investigated the association between *TIMP* gene polymorphisms and chronic HF. Therefore, we tested the hypothesis that the 372 T > C polymorphism (Phe124Phe; rs4898) in the *TIMP-1* gene and the −418 G > C polymorphism (rs8179090) in the *TIMP-2* gene are associated with HF susceptibility, all-cause death, and/or HF-related death in Brazilians with reduced LV ejection fraction (LVEF).

## Results

### Association of the 372 T > C polymorphism with HF susceptibility and clinical profile

The genotype frequencies were in agreement with those predicted by the Hardy-Weinberg equilibrium for the 372 T > C polymorphism in the *TIMP-1* gene in both HF patients and healthy blood donors. The genotype and allele frequencies in HF patients were not significantly different from those observed in blood donors (Table 1). The frequencies of the T and C alleles also did not differ between male and female HF patients (*P* = 0.561) or blood donors (*P* = 0.907). Regarding the −418 G > C polymorphism in the *TIMP-2* gene, only five heterozygous subjects (GC) were found among the 263 HF patients (*n* = 4) and 260 blood donors (*n* = 1) genotyped for this polymorphism. The four heterozygous patients were males and the heterozygous blood donor was a female. All the other subjects were homozygotes for the G allele. Thus, this gene variant could not be further analysed.

**Table 1:** Comparison of genotype and allele frequencies of the 372 T > C polymorphism between healthy blood donors and heart failure (HF) patients stratified by gender.

	Blood donors (n = 304)	HF patients (n = 300)	P*
**Females**			
Genotype	n = 93	n = 99	
TT, n (%)	26 (28.0)	39 (39.4)	0.226
TC, n (%)	47 (50.5)	40 (40.4)	
CC, n (%)	20 (21.5)	20 (20.2)	
Allele	n = 186	n = 198	
T, n (%)	99 (53.2)	118 (59.6)	0.248
C, n (%)	87 (46.8)	80 (40.4)	
Males	n = 211	n = 201	
T, n (%)	110 (52.1)	113 (56.2)	0.463
C, n (%)	101 (47.9)	88 (43.8)	

*P-values were calculated using the Pearson chi-square test with Yates correction where appropriate.

Subjects included in this study were predominantly male (69.4% and 67.0% of blood donors and HF patients, respectively; *P* = 0.584) and white (78.3% and 71.3% of blood donors and HF patients, respectively; *P* = 0.061). Blood donors were younger than HF patients (mean age of 48 ± 10 and 60 ± 13, respectively; *P* < 0.001). The baseline demographic and clinical characteristics of HF patients according to the presence of the 372 T allele in the *TIMP-1* gene are shown in Table 2. Patients were in New York Heart Association (NYHA) class I or II and had predominantly ischaemic aetiology and moderate to severe left ventricular dysfunction. Approximately one-third of the patients had comorbidities such as prior AMI and diabetes mellitus.

**Table 2:** Comparison of the baseline profile of HF patients with and without the T allele of the 372 T > C polymorphism.

	All patients (n = 300)	CC + C (n = 108)	TT + TC + T (n = 192)	P*
Age (years, mean ± SD)	60 ± 13	59 ± 12	60 ± 13	0.424
White, n (%)	214 (71.3)	73 (67.6)	141 (73.4)	0.346
**HF aetiology**				
Ischaemic, n (%)	109 (36.3)	41 (38.0)	68 (35.4)	0.753
Idiopathic, n (%)	88 (29.3)	30 (27.8)	58 (30.2)	0.755
Hypertensive, n (%)	72 (24.0)	23 (21.3)	49 (25.5)	0.496
NYHA classes I and II, n (%)^†^	231 (78.3)	85 (79.4)	146 (77.7)	0.834
Prior myocardial infarction, n (%)	97 (32.3)	32 (29.6)	65 (33.9)	0.534
History of smoking, n (%)	139 (46.3)	56 (51.9)	83 (43.2)	0.188
Diabetes mellitus, n (%)	90 (30.0)	32 (29.6)	58 (30.2)	>0.999
**Electrocardiogram**				
QRS duration (ms, mean ± SD)	129 ± 35	127 ± 34	130 ± 35	0.664
Atrial fibrillation, n (%)^‡^	71 (24.1)	28 (26.2)	43 (23.0)	0.638
Left bundle branch block, n (%)^§^	84 (28.7)	22 (20.8)	62 (33.2)	**0.034**
Right bundle branch block, n (%)^§^	16 (5.5)	8 (7.5)	8 (4.3)	0.360
**Echocardiography**				
LVEF (%, mean ± SD)	31 ± 8	32 ± 8	31 ± 8	0.537
LVEDD (mm, mean ± SD)	6.6 ± 0.9	6.7 ± 0.9	6.6 ± 0.9	0.185
LVESD (mm, mean ± SD)	5.6 ± 1.0	5.7 ± 1.0	5.5 ± 1.0	0.084
Creatinine (µmol/L, mean ± SD)	113 ± 40	114 ± 42	112 ± 39	0.796
Urea (mmol/L, median [25^th^–75^th^ percentiles])	17 [13−24]	15 [12−22]	18 [14−27]	**0.019**
Sodium (mEq/L, mean ± SD)	140 ± 3	140 ± 3	140 ± 4	0.270
Haemoglobin (g/dL, mean ± SD)	13.1 ± 1.7	13.3 ± 1.8	13.0 ± 1.6	0.209
**HF medications**				
Beta-blocker, n (%)	266 (88.7)	97 (89.8)	169 (88.0)	0.779
ACE inhibitor, n (%)	263 (87.7)	96 (88.9)	167 (87.0)	0.764

Abbreviations: HF, heart failure; NYHA, New York Heart Association; LVEF, left ventricular ejection fraction; LVEDD, left ventricular end-diastolic diameter; LVESD, left ventricular end-systolic diameter; and ACE, angiotensin-converting enzyme. *P-values were calculated using the Mann-Whitney U test for continuous variables and the Pearson chi-square test for categorical variables, with Yates correction where appropriate. ^†^Data available for 107 and 188 patients without and with the T allele, respectively. ^‡^Data available for 107 and 187 patients without and with the T allele, respectively. ^§^Data available for 106 and 187 patients without and with the T allele, respectively.

In general, carriers of the 372 T allele had a similar clinical profile to that of female homozygotes and male hemizygotes for the C allele. However, subjects with the T allele more often had left bundle branch block and higher blood urea levels than females with the CC genotype or males with the C allele (Table 2). Considering the left bundle branch block as an outcome, we observed that the T allele was more frequent in males with left bundle branch block than in those who did not have this conduction disorder (see Supplementary Table S1). After controlling for skin colour (white/non-white), age, LVEF, haemoglobin level, and jugular venous pressure, the T allele did not remain associated with left bundle branch block in the multiple logistic regression analysis (adjusted odds ratio, 1.91; 95% confidence interval, 0.86−4.28; *P* = 0.114).

### Association of the 372 T > C polymorphism with HF prognosis

**Figure 1:**
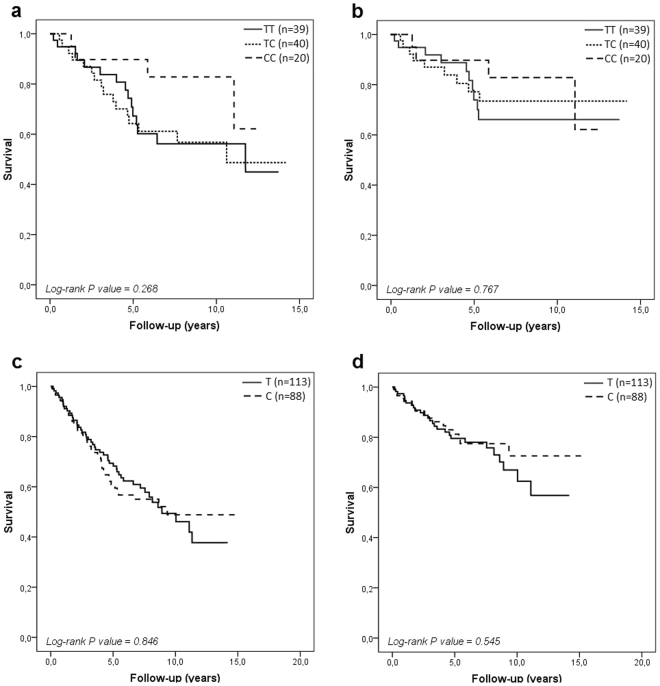
Survival curves for females (**a** and **b**) and males (**c** and **d**) for all-cause death and HF-related death, respectively, according to the 372 T > C polymorphism in the *TIMP-1* gene.

Patients were followed-up for a median duration of approximately 5.5 years (ranging from 1 month to 15 years). During this period, 121 patients (40.3%) died (67 of them from HF). The overall mortality rate was not different between males and females (42.8% vs. 35.4%, respectively; *P* = 0.268). Figure 1 shows the survival curves for all-cause death (A and B) and HF-related death (C and D) in females and males according to the 372 T > C polymorphism in the *TIMP-1* gene. Although females who carried the T allele had a worse prognosis than those who were homozygous for the C allele (Fig. 1a), this difference in survival was not significantly different. Among males, survival curves were quite similar between patients hemizygous for the T and C alleles (Fig. 1b and d).

## Discussion

In this study, we evaluated the 372 T > C (Phe124Phe) polymorphism in the *TIMP-1* gene and the −418 G > C polymorphism in the *TIMP-2* gene in 300 HF patients with reduced LVEF and 304 healthy blood donors from the State of Rio Grande do Sul (Southern Brazil). We did not find an association between the 372 T > C polymorphism in the *TIMP-1* gene and HF susceptibility or mortality among HF patients. Moreover, the presence of the C nucleotide at position −418 in the *TIMP-2* gene was very rare in our population.

Although several studies have reported that increased expression of TIMP-1 is associated with echocardiographic parameters of LV systolic dysfunction^28–33^ and worse prognosis in HF patients^30,31,33–38^, to the best of our knowledge, no study has investigated the association between gene polymorphisms of *TIMPs* and chronic HF. In the context of HF, Goldbergova *et al*.^23^ evaluated the relationship of gene polymorphisms and serum levels of TIMP-1 with AMI, LV dysfunction and symptoms of acute HF in patients with ST elevation treated with primary percutaneous coronary intervention in the Czech population (Caucasians). TIMP-1 levels were higher in males with acute HF as well as in males with reduced LVEF (<40%). However, TIMP-1 levels did not differ depending on the 372 T > C polymorphism, and this polymorphism was not independently associated with LV dysfunction post-AMI (acute HF) after adjusting for clinical and laboratory covariates^23^. In a more recent report from Goldbergova *et al*.^24^ in the same study population, there was no evidence of association between the 372 T > C polymorphism and a composite endpoint, including hospitalisation due to acute decompensated HF and all causes of death, in the cohort followed-up for a median of 32 months.

In our study, genotype and allele frequencies of the 372 T > C polymorphism were not different between HF patients and healthy blood donors. Similarly, most of the clinical, electrocardiographic, echocardiographic, and laboratory parameters did not differ between carriers of the T allele and subjects without the T allele. These findings indicate that the 372 T > C polymorphism is not associated with the occurrence of HF or AMI (clinical event previously suffered by one-third of the patients), which is also in accordance with the results reported by Horne *et al*.^22^. The North-American study evaluated the association of different polymorphisms in seven genes encoding MMPs and TIMPs -1, -2, and -3 with AMI in more than 5000 patients (92% Caucasians) who underwent coronary angiography in tertiary hospitals in Utah. No association of the 372 T > C polymorphism with AMI or CAD was detected in that population^22^.

Regarding HF prognosis, considering that TIMP-1 levels are increased in patients with systolic dysfunction and worse prognosis and the T allele of the 372 T > C polymorphism is associated with increased TIMP-1 levels, the T allele is expected to be associated with HF susceptibility or a higher mortality rate among HF patients. In our study, the survival analysis showed that female carriers of the T allele seemed to have a higher rate of all-cause death than females homozygous for the C allele, mainly between the sixth and the tenth year of follow-up. However, the difference in survival according to the 372 T > C genotype was not statistically significant. Considering that the frequency of the T allele carriers was 63% among patients who were alive at the end of follow-up (males and females), our study had a statistical power of 79.6% to detect an odds ratio = 2.08 related to all-cause death, as reported by Lorente *et al*.^21^ in patients with severe sepsis.

The T allele of the 372 T > C polymorphism, which is possibly associated with increased levels of TIMP-1 and worse prognosis, showed a frequency of 53% in the general population (blood donors) from Rio Grande do Sul, which is identical to the frequency detected in the 1000 Genomes Project (database available at https://www.ncbi.nlm.nih.gov/projects/SNP/snp_ref.cgi?rs=4898). Moreover, genotype and allele frequencies of the 372 T > C polymorphism did not differ between white and non-white HF patients or blood donors in our study (see Supplementary Table S2). Regarding the −418 G > C polymorphism in the *TIMP-2* gene, the rarity of the C allele in our study population (frequency of 0.8% among HF patients and 0.2% among blood donors) is in accordance with the frequency observed in South-eastern Brazilians without Japanese descent and Europeans^39^, in whom the frequency of the C allele is 0.5% (https://www.ncbi.nlm.nih.gov/projects/SNP/snp_ref.cgi?rs=8179090).

Because it is located on the X chromosome^10^, it is expected that the *TIMP-1* gene would have the same level of expression in both males and females due to the X chromosome inactivation phenomenon. However, cell culture studies have shown that the *TIMP-1* gene is one of the human genes that escape from X inactivation and is expressed in both the active and inactive X chromosomes, although expression from the latter allele is more variable and may be lower than from the former allele^40^. Moreover, inactivation of the *TIMP-1* gene is variable (polymorphic), as some females express both alleles, whereas in others, only the allele in the active chromosome is expressed, which can lead to differences in TIMP-1 expression among homozygous and heterozygous females^41^.

Protein levels are also influenced by the transcription rate, mRNA stability, and translation^42,43^. Even though it is a silent (synonymous) variant, a functional effect of the 372 T > C polymorphism (Phe124Phe) on the activity of TIMP-1 in the myocardium cannot be ruled out. Although the synonymous mutation has no effect on the sequence of the synthesised protein, its presence can lead to changes in expression, conformation, or substrate specificity, thereby impacting the physiological function of the protein. Approximately 40 diseases affecting different organs and systems have been associated with synonymous mutations. Experimental studies and computational predictions show that there are different mechanisms by which a synonymous mutation can affect the synthesis of an active and correctly folded protein. These mechanisms include changes in the splicing pattern (exon skipping), mRNA secondary structure and stability, and the translation rate due to codon usage bias and altered microRNA binding sites^42^. As the functionality of the 372 T > C polymorphism has not yet been tested, it is unknown how this variant would affect the synthesis of TIMP-1. In addition, the 372 T > C polymorphism may be in linkage disequilibrium with other variants in the same gene whose function may be affected in the presence of two or more synonymous mutations^42,44^.

Our findings should be interpreted with consideration of some limitations. The retrospective design (case-control) of our study regarding HF susceptibility does not allow us to examine whether the 372 T > C polymorphism in the *TIMP-1* gene influences LV remodelling and HF onset or which allele would be involved in these processes. Due to our focus on systolic dysfunction, we did not collect data on diastolic function of left ventricle, which is another limitation of this study. Although our study had sufficient statistical power for the analysis of mortality as an outcome, the reduced sample size did not allow us to analyse potential modifiers that might contribute to HF variability, such as age and cardiomyopathy aetiology. Apart from this, quantification of the serum levels of TIMP-1 would also help to clarify the functionality of the 372 T > C polymorphism or, at least, the link between this gene variant and TIMP-1 expression. This is the major limitation of our study, as the relationship of the 372 T > C polymorphism with TIMP-1 levels is still doubtful and there is no clear evidence showing this link. Despite all limitations, this was the first study to investigate a polymorphism in the *TIMP-1* gene in a cohort of patients with chronic HF who underwent long-term follow-up (up to 15 years). In conclusion, the 372 T > C polymorphism (Phe124Phe; rs4898) in the *TIMP-1* gene and the −418 G > C polymorphism (rs8179090) in the *TIMP-2* gene are associated with neither HF susceptibility nor survival in HF patients with reduced LVEF in a population from South Brazil.

## Methods

### Study population

A total of 300 adults with HF of any aetiology and a reduced LVEF (≤45%) were included in this study. HF was diagnosed according to the ACCF/AHA guidelines^45^, and all patients were enrolled consecutively between July 2003 and November 2007 in the Heart Failure and Transplant Outpatient Clinic of a tertiary care university hospital (HCPA) in Porto Alegre, Brazil. Patients underwent a comprehensive clinical and laboratory evaluation consisting of a physical examination, assessment of electro- and echocardiographic parameters, and laboratory exams at baseline. Briefly, two-dimensional transthoracic echocardiography with M-mode and Doppler examination was performed using an adult transducer and a commercial ultrasound system (Philips EnVisor, Andover, USA). Echocardiography was performed by experienced staff cardiologists in the non-invasive cardiac unit of HCPA, as part of the routine management of HF patients, following the recommendations of the American Society of Echocardiography for LVEF estimations^46^. Ischaemic aetiology was defined as previously described^18^. A questionnaire was used to collect data on medical history, such as the age at HF diagnosis, smoking habits, presence of comorbidities, and use of medication. Patients were followed in the outpatient clinic at our institution, and their vital status was assessed using hospital records, by telephone contact, or using the State Death Certificate Database. The prognostic endpoints of interest were all-cause death and HF-related death, which was defined as sudden unexpected death (within 1 h of symptom onset) or death caused by advanced refractory disease. Survival data were last updated on April 2014.

We also enrolled 304 unrelated blood donors in the same hospital during the same period as the HF patients. Subjects with a history of cardiovascular disease or related symptoms and those with a positive family history of premature sudden death were not included in the study, and no additional laboratory data were collected from them. Skin colour/ethnicity of all subjects was self-reported and categorized as white or non-white (pardo or black). All experiments were performed in accordance with the relevant guidelines and regulations. The study was approved by the hospital ethics committee (Institutional Review Board [IRB] 0000921 – application number 03–237), and all subjects provided written informed consent prior to their participation.

### Genotyping

Genomic DNA was isolated from peripheral blood leukocytes using the salting out procedure described by Lahiri & Nurnberger^47^. Genotypes of the 372 T > C polymorphism in the *TIMP-1* gene were determined by real-time polymerase chain reaction (PCR) using the probes and primers provided in the Human Custom TaqMan Genotyping Assay (forward: 5′-GGACTCTTGCACATCACTACCT-3′ and reverse: 5′-GCTAAGCTCAGGCTGTTCCA-3′; Life Technologies, Carlsbad, USA). The probe for the C allele was labelled with FAM dye (5′-AGCCACGAAACTG-3′), while the probe for the T allele was labelled with VIC dye (5′-AGCCACAAAACTG-3′). Amplification reactions were performed in optical tubes in a total volume of 10 µL, containing 2 ng of genomic DNA, 1× TaqMan Genotyping Master Mix, and the 1× genotyping assay. Optical tubes were loaded into a real-time PCR thermal cycler (StepOnePlus; Life Technologies) and heated at 95 °C for 10 min followed by 40 cycles of denaturation at 95 °C for 15 s and annealing/extension at 63 °C for 1 min. Fluorescence data from each run were analysed with the StepOne software version 2.3 (Life Technologies).

Genotypes of the −418 G > C polymorphism in the *TIMP-2* gene were determined by the PCR and restriction fragment length polymorphism (PCR-RFLP) method using the primers previously described by Yi *et al*.^48^ in the amplification reactions and the *BsoB*I restriction enzyme to digest the amplified fragments (New England Biolabs, Ipswich, USA).

Amplification reactions were done in a total volume of 25 μL containing 50 to 100 ng DNA, 0.3 μM each primer, 1× PCR buffer, 1.5 mM MgCl_2_, 200 mM each dNTP, and 1 U *Taq* DNA polymerase (Fermentas Life Sciences, Burlington, USA). After the initial denaturation step for 3 min at 94 °C, the reaction mixture was subjected to 35 cycles of 40 s at 94 °C, 40 s at 63 °C, and 40 s at 72 °C, followed by a final extension step of 3 min at 72 °C. PCR products were subjected to overnight digestion with 5 U *BsoB*I following the manufacturer’s instructions (New England Biolabs, Ipswich, USA). The 304-bp amplicon with the C allele is cleaved into fragments of 253 and 51 bp, whereas the G allele creates an additional restriction site, thus generating fragments of 230, 51 and 23 bp. The resulting digested fragments were electrophoresed on 8% polyacrylamide gels and then stained with silver nitrate. To improve genotyping accuracy, a DNA sample of each genotype was used in all runs to serve as a positive control, and the investigators who independently identified the genotypes (E.R.P. and R.C.S.) were blinded to the clinical and laboratory data of the patients. However, we did not repeat the genotyping in a randomly chosen subgroup of subjects.

### Statistical analyses

Continuous data are expressed as the mean ± standard deviation (SD) or median (25^th^–75^th^ percentiles) and were compared between the groups using the Mann-Whitney U test. The Shapiro-Wilk test was used to verify the normality of quantitative variables. Categorical variables, including the genotype and allele frequencies, are expressed as an absolute frequency (percentage) and were compared between groups with the Pearson chi-square test; the Yates correction was applied for the comparisons involving 2 × 2 contingency tables. Allele frequencies were determined by gene counting, and departures from Hardy-Weinberg equilibrium were also verified using the chi-square test.

The association of the 372 T > C polymorphism in the *TIMP-1* gene with all-cause and HF-related death was evaluated by Kaplan-Meier survival analysis. Survival curves were constructed based on the period between the date of the first visit at the outpatient clinic and the last registry of follow-up or death. Survival curves obtained for the different genotypes were compared by the log-rank test. Wherever needed, logistic regression analysis was used to assess the association of the 372 T > C polymorphism with the clinical profile of HF patients. Statistical analyses were performed using SPSS (version 18.0; SPSS Inc., Chicago, USA) and WinPEPI (version 11.43)^49^ statistical software. Two-tailed *P-*values < 0.05 were considered statistically significant.

### Data availability

Relevant raw data are included in the Supplementary Information files (see Supplementary Data). Age, skin colour, and other variables that could identify the study participants were omitted to protect their confidentiality and privacy.

## Electronic supplementary material

Supplementary Tables

Dataset 1

## References

[1] Bloom MW (2017). Heart failure with reduced ejection fraction. Nat Rev Dis Primers..

[2] Guo M, Guo G, Ji X (2016). Genetic polymorphisms associated with heart failure: a literature review. J Int Med Res..

[3] Burchfield JS, Xie M, Hill JA (2013). Pathological ventricular remodeling: mechanisms: part 1 of 2. Circulation..

[4] Suthahar N, Meijers WC, Silljé HHW, de Boer RA (2017). From inflammation to fibrosis-molecular and cellular mechanisms of myocardial tissue remodelling and perspectives on differential treatment opportunities. Curr Heart Fail Rep..

[5] DeLeon-Pennell KY, Meschiari CA, Jung M, Lindsey ML (2017). Matrix metalloproteinases in myocardial infarction and heart failure. Prog Mol Biol Transl Sci..

[6] Spinale FG (2007). Myocardial matrix remodeling and the matrix metalloproteinases: influence on cardiac form and function. Physiol Rev..

[7] Vanhoutte D, Heymans S (2010). TIMPs and cardiac remodeling: ‘Embracing the MMP-independent-side of the family’. J Mol Cell Cardiol..

[8] Ky B (2013). Ventricular-arterial coupling, remodeling, and prognosis in chronic heart failure. J Am Coll Cardiol..

[9] Moore L (2012). Tissue inhibitor of metalloproteinases (TIMPs) in heart failure. Heart Fail Rev..

[10] Ries C (2014). Cytokine functions of TIMP-1. Cell Mol Life Sci..

[11] Marchesi C (2012). Plasma levels of matrix metalloproteinases and their inhibitors in hypertension: a systematic review and meta-analysis. J Hypertens..

[12] Schanz M, Shi J, Wasser C, Alscher MD, Kimmel M (2017). Urinary [TIMP-2] × [IGFBP7] for risk prediction of acute kidney injury in decompensated heart failure. Clin Cardiol..

[13] Nanni S (2007). Matrix metalloproteinases in premature coronary atherosclerosis: influence of inhibitors, inflammation, and genetic polymorphisms. Transl Res..

[14] Wilson EM (2002). Plasma matrix metalloproteinase and inhibitor profiles in patients with heart failure. J Card Fail..

[15] Stanciu AE (2013). Cardiac resynchronization therapy in patients with chronic heart failure is associated with anti-inflammatory and anti-remodeling effects. Clin Biochem..

[16] Lin TH (2014). Mitral tissue inhibitor of metalloproteinase 2 is associated with mitral valve surgery outcome. PLoS One..

[17] Silvello D (2014). Serum levels and polymorphisms of matrix metalloproteinases (MMPs) in carotid artery atherosclerosis: higher MMP-9 levels are associated with plaque vulnerability. Biomarkers..

[18] Velho FM (2011). Polymorphisms of matrix metalloproteinases in systolic heart failure: role on disease susceptibility, phenotypic characteristics, and prognosis. J Card Fail..

[19] Beber AR (2016). Matrix metalloproteinase-2 polymorphisms in chronic heart failure: relationship with susceptibility and long-term survival. PLoS One..

[20] Ho WM (2015). Association of MMP-9 haplotypes and TIMP-1 polymorphism with spontaneous deep intracerebral hemorrhage in the Taiwan population. PLoS One..

[21] Lorente L (2013). The 372 T/C genetic polymorphism of TIMP-1 is associated with serum levels of TIMP-1 and survival in patients with severe sepsis. Crit Care..

[22] Horne BD (2007). Multiple-polymorphism associations of 7 matrix metalloproteinase and tissue inhibitor metalloproteinase genes with myocardial infarction and angiographic coronary artery disease. Am Heart J..

[23] Goldbergova MP (2012). The association between levels of tissue inhibitor of metalloproteinase-1 with acute heart failure and left ventricular dysfunction in patients with ST elevation myocardial infarction treated by primary percutaneous coronary intervention. Genet Test Mol Biomarkers..

[24] Goldbergova MP (2017). Relationship of long-term prognosis to MMP and TIMP polymorphisms in patients after ST elevation myocardial infarction. J Appl Genet..

[25] Hirano K (2001). Tissue inhibitor of metalloproteinases-2 gene polymorphisms in chronic obstructive pulmonary disease. Eur Respir J..

[26] Lin TH (2007). The C-allele of tissue inhibitor of metalloproteinases 2 is associated with increased magnitude of QT dispersion prolongation in elderly Chinese - 4-year follow-up study. Clin Chim Acta..

[27] Gai X (2010). MMP-2 and TIMP-2 gene polymorphisms and susceptibility to atrial fibrillation in Chinese Han patients with hypertensive heart disease. Clin Chim Acta..

[28] Sundström J (2004). Relations of plasma total TIMP-1 levels to cardiovascular risk factors and echocardiographic measures: the Framingham Heart Study. Eur Heart J..

[29] Picard F (2006). Increased cardiac mRNA expression of matrix metalloproteinase-1 (MMP-1) and its inhibitor (TIMP-1) in DCM patients. Clin Res Cardiol..

[30] Frantz S (2008). Tissue inhibitor of metalloproteinase levels in patients with chronic heart failure: an independent predictor of mortality. Eur J Heart Fail..

[31] Jungbauer CG (2014). Panel of emerging cardiac biomarkers contributes for prognosis rather than diagnosis in chronic heart failure. Biomark Med..

[32] Maharaj N (2014). Relationship between left ventricular twist and circulating biomarkers of collagen turnover in hypertensive patients with heart failure. J Am Soc Echocardiogr..

[33] Sanchis L (2015). Prognosis of new-onset heart failure outpatients and collagen biomarkers. Eur J Clin Invest..

[34] Barton PJ (2003). Increased expression of extracellular matrix regulators TIMP1 and MMP1 in deteriorating heart failure. J Heart Lung Transplant..

[35] Franz M (2013). Matrix metalloproteinase-9, tissue inhibitor of metalloproteinase-1, B^+^ tenascin-C and ED-A^+^ fibronectin in dilated cardiomyopathy: potential impact on disease progression and patients’ prognosis. Int J Cardiol..

[36] Chang YY (2014). Comparison the prognostic value of galectin-3 and serum markers of cardiac extracellular matrix turnover in patients with chronic systolic heart failure. Int J Med Sci..

[37] Trucco E (2016). Plasma tissue inhibitor of matrix metalloproteinase-1 a predictor of long-term mortality in patients treated with cardiac resynchronization therapy. Europace..

[38] Morishita T (2017). Association between matrix metalloproteinase-9 and worsening heart failure events in patients with chronic heart failure. ESC Heart Fail..

[39] da Silva RA (2013). *TIMP2* gene polymorphism as a potential tool to infer Brazilian population origin. Adv Genomics Genet..

[40] Anderson CL, Brown CJ (2002). Variability of X chromosome inactivation: effect on levels of TIMP1 RNA and role of DNA methylation. Hum Genet..

[41] Anderson CL, Brown CJ (1999). Polymorphic X-chromosome inactivation of the human TIMP1 gene. Hum Genet..

[42] Sauna ZE, Kimchi-Sarfaty C (2011). Understanding the contribution of synonymous mutations to human disease. Nat Rev Genet..

[43] Strachan, T. & Read, A. *Human molecular genetics* (Garland Science/Taylor & Francis Group, 2011).

[44] Fung KL, Gottesman MM (2009). A synonymous polymorphism in a common MDR1 (ABCB1) haplotype shapes protein function. Biochim Biophys Acta..

[45] Jessup M (2009). 2009 focused update: ACCF/AHA guidelines for the diagnosis and management of heart failure in adults: a report of the American College of Cardiology Foundation/American Heart Association task force on practice guidelines developed in collaboration with the International Society for Heart and Lung Transplantation. J Am Coll Cardiol..

[46] Lang RM (2005). Recommendations for chamber quantification: a report from the American Society of Echocardiography’s Guidelines and the Standards Committee and the Chamber Quantification Writing Group, developed in conjunction with the European Association of Echocardiography, a branch of the European Society of Cardiology. J Am Soc Echocardiogr..

[47] Lahiri DK, Nurnberger JI (1991). A rapid non-enzymatic method for the preparation of HMW DNA from blood for RFLP studies. Nucleic Acids Res..

[48] Yi YC (2009). Genetic polymorphism of the tissue inhibitor of metalloproteinase-1 is associated with an increased risk of endometrial cancer. Clin Chim Acta..

[49] Abramson JH (2011). WINPEPI updated: computer programs for epidemiologists, and their teaching potential. Epidemiol Perspect Innov..

