# Supplementary material for: Relationship of polymorphisms in the tissue inhibitor of metalloproteinase (TIMP)-1 and -2 genes with chronic heart failure
Source: Sci Rep. 2018 Jun 21;8:9446. doi: 10.1038/s41598-018-27857-5 (PMC6013444; doi:10.1038/s41598-018-27857-5)
Supplement: Supplementary file 1 — Supplementary Tables [file 41598_2018_27857_MOESM1_ESM.pdf]

Relationship of polymorphisms in the tissue inhibitor of metalloproteinase (*TIMP*)-1 and -2 genes with chronic heart failure

Evelise Regina Polina[1], Raquel Rosa Candebat Vallejo Araújo[1], Renan Cesar Sbruzzi[1], Andréia Biolo[2,3], Luís Eduardo Rohde[2,3], Nadine Clausell[2,3] & Kátia Gonçalves dos Santos[1,2,*]

[1]Laboratory of Human Molecular Genetics, Universidade Luterana do Brasil, Canoas, 92425-900, Brazil

[2]Heart Failure and Cardiac Transplant Unit, Cardiology Division, Hospital de Clínicas de Porto Alegre (HCPA), Porto Alegre, 90035-903, Brazil

[3]Department of Internal Medicine, Medical School, Universidade Federal do Rio Grande do Sul (UFRGS), Porto Alegre, 90040-341, Brazil

*Corresponding author: kgsantos2010@gmail.com

Supplementary Table S1. Comparison of genotype and allele frequencies of the 372T>C polymorphism between heart failure patients with and without left bundle branch block (LBBB).

	**Without LBBB (n = 209)**	**With LBBB (n = 84)**	***P****
Females			
Genotype	n = 63	n = 36	
TT, n (%)	25 (39.7)	14 (38.9)	0.979
TC, n (%)	25 (39.7)	15 (41.7)	
CC, n (%)	13 (20.6)	7 (19.4)	
Allele	n = 126	n = 72	
T, n (%)	75 (59.5)	43 (59.7)	>0.999
C, n (%)	51 (40.5)	29 (40.3)	
Males	n = 146	n = 48	
T, n (%)	75 (51.4)	33 (68.7)	0.053
C, n (%)	71 (48.6)	15 (31.3)	

**P*-values were calculated using the Pearson chi-square test with Yates correction where appropriate. Left bundle branch block data were available for 293 patients (instead of 300).

Supplementary Table S2. Comparison of genotype and allele frequencies of the 372T>C polymorphism between white and non-white subjects stratified by clinical status.

	**Blood donors (n = 304)**			**Heart failure patients (n = 300)**		
	**White (n = 238)**	**Non-white (n = 66)**	***P****	**White (n = 214)**	**Non-white (n = 86)**	***P****
Females						
Genotype	n = 76	n = 17		n = 74	n = 25	
TT, n (%)	20 (26.3)	6 (35.3)	0.672	29 (39.2)	10 (40.0)	0.818
TC, n (%)	40 (52.6)	7 (41.2)		29 (39.2)	11 (44.0)	
CC, n (%)	16 (21.1)	4 (23.5)		16 (21.6)	4 (16.0)	
Allele	n = 152	n = 34		n = 148	n = 50	
T, n (%)	80 (52.6)	19 (55.9)	0.878	87 (58.8)	31 (62.0)	0.815
C, n (%)	72 (47.4)	15 (44.1)		61 (41.2)	19 (38.0)	
Males	n = 162	n = 49		n = 140	n = 61	
T, n (%)	87 (53.7)	23 (46.9)	0.504	83 (59.3)	30 (49.2)	0.241
C, n (%)	75 (46.3)	26 (53.1)		57 (40.7)	31 (50.8)	

**P*-values were calculated using chi-square test with Yates correction where appropriate.
